# Vitamin D supplementation in cutaneous malignant melanoma outcome (ViDMe): a randomized controlled trial

**DOI:** 10.1186/s12885-017-3538-4

**Published:** 2017-08-23

**Authors:** J. De Smedt, S. Van Kelst, V. Boecxstaens, M. Stas, K. Bogaerts, D. Vanderschueren, C. Aura, K. Vandenberghe, D. Lambrechts, P. Wolter, O. Bechter, A. Nikkels, T. Strobbe, G. Emri, V. Marasigan, M. Garmyn

**Affiliations:** 1 Laboratory of Dermatology, Department of oncology, KU Leuven, 3000 Leuven, Belgium; 2 Department of Dermatology, University Hospitals Leuven, 3000 Leuven, Belgium; 3 Oncological and vascular access surgery, Department of surgical oncology, University Hospitals Leuven, 3000 Leuven, Belgium; 4 Department of oncology, KU Leuven, 3000 Leuven, Belgium; 5 KU Leuven, Faculty of Medicine, I-BioStat, 3000 Leuven, Belgium; 6 Hasselt University, I-BioStat, 3590 Diepenbeek, Belgium; 7 Clinical and Experimental Endocrinology, Department of Clinical and Experimental Medicine, KU Leuven, 3000 Leuven, Belgium; 8 Department of Endocrinology, University Hospitals Leuven, 3000 Leuven, Belgium; 9 Translational Cell & Tissue Research, Department of Imaging & Pathology, KU Leuven, 3000 Leuven, Belgium; 10 Department of Pathology, University Hospitals of Leuven, 3000 Leuven, Belgium; 11 Department of Cardiovascular Sciences, KU Leuven, 3000 Leuven, Belgium; 12 Laboratory for Translational Genetics, Department of Oncology, KU Leuven, 3000 Leuven, Belgium; 13 Vesalius Research Center, VIB, 3000 Leuven, Belgium; 14 Department of Hematology and Oncology, CHR Verviers East Belgium, 4800 Verviers, Belgium; 15 Laboratory of Experimental Oncology (LEO), Department of Oncology, KU Leuven, 3000 Leuven, Belgium; 16 Department of General Medical Oncology, University Hospitals Leuven, Leuven Cancer Institute, 3000 Leuven, Belgium; 17 Department of Dermatology, CHU Sart Tilman, University of Liège, 4000 Liège, Belgium; 18 Department of Dermatology, University Hospital Antwerp, 2650 Edegem, Belgium; 19 Department of Dermatology, Faculty of Medicine, University of Debrecen, Debrecen, 4012 Hungary

**Keywords:** Melanoma, Vitamin D, Randomized controlled trial, Safety, Secondary prevention

## Abstract

**Background:**

Previous studies have investigated the protective effect of vitamin D serum levels, at diagnosis and during the follow-up period after treatment, on melanoma outcome. In the present study we assess whether vitamin D supplementation, in the follow-up period after diagnosis and surgical resection of the primary tumor, has a protective effect on relapse of cutaneous malignant melanoma and whether this protective effect correlates with vitamin D levels in serum and Vitamin D Receptor immunoreactivity in the primary tumor.

**Methods/design:**

This study is a multicenter randomized double blind placebo- controlled phase III trial. Patients between the age of 18 and 80 years diagnosed and treated surgically for a melanoma stage IB-III are eligible for randomization in a 1:1 ratio to active treatment or placebo. The study drug is taken each month and consists of either 100,000 International Unit cholecalciferol or arachidis oleum raffinatum used as a placebo. The primary endpoint is relapse free survival. The secondary endpoints are 25 hydroxyvitamin D3 serum levels at diagnosis and at 6 month intervals, melanoma subtype, melanoma site and stage of melanoma at diagnosis according to the 2009 American Joint Committee on Cancer melanoma staging and classification. At randomization a bloodsample is taken for DNA analysis. The study is approved by the local Ethics Committees.

**Discussion:**

If we can confirm our hypothesis that vitamin D supplementation after removal of the tumor has a protective effect on relapse of cutaneous malignant melanoma we may reduce the burden of CMM at several levels. Patients, diagnosed with melanoma may have a better clinical outcome and improved quality of life. There will be a decrease in health care costs related to treatment of metastatic disease and there will be a decrease in loss of professional years, which will markedly reduce the economic burden of the disease.

**Trial registration:**

Clinical Trial.gov, NCT01748448, 05/12/2012

## Background

### Cutaneous malignant melanoma: risk factors for development and progression, current knowledge

Cutaneous malignant melanoma (CMM) is the 7th most frequent tumor in males and 4th most frequent tumor in females in Belgium [1]. It only accounts for 4% of all malignant tumors of the skin, but it is responsible for 80% of the skin cancer related deaths [2, 3].

In 2013 the Belgian CMM incidence was 19.9 cases per 100,000 men (Crude Rate) and 18.5 cases per 100,000 women (Crude Rate) [4]. During the last decade’s incidence rates have been rising in many countries within white populations and it is expected that the incidence will further increase the coming years [5, 6]. CMM arises from a stepwise transformation of melanocytes in the skin, with subsequent superficial and deep invasion ultimately forming metastasis [3]. Several risk factors have been linked to the development of CMM. The strongest risk factors for melanoma are a family history of melanoma, multiple benign or atypical naevi and a previous melanoma. Immunosuppression, low phototype and exposure to ultraviolet light are additional risk factors [7]. The most important clinicopathological subtypes of melanoma are superficial spreading malignant melanoma, nodular malignant melanoma, lentigo maligna melanoma and acrolentiginous malignant melanoma. Diagnostic signs are progressive asymmetrical enlargement of a pigment lesion which also demonstrates irregularity in colour and irregularity in shape. Different risk factors are associated with different subtypes of CMM and different locations of malignant melanoma [8]. Different subtypes of malignant melanomas show also a significant genetic heterogeneity. The most important predictor for melanoma relapse and melanoma specific survival is Breslow thickness of the primary tumor at diagnosis [9]. This is a histological characteristic of the tumor and refers to the thickness of the tumor, measured as the distance between the granular layer of the epidermis and the deepest tumor cell in millimeters. Additional independent prognostic factors for survival are primary tumor ulceration and primary tumor mitotic rate according to the 2009 American Joint Committee on Cancer (AJCC) melanoma classification. Other known predictive prognostic factors are presence and distribution of tumor infiltrating lymphocytes, tumor site, sex and social deprivation [9, 10]. The overall survival of CMM is better for earlier stages of the disease, however relapse poses a significant threat even for patients with limited disease. For this patient population we need an adjuvant treatment strategy which is cost/efficient, feasible and safe. Vitamin D supplementation is such a potential adjuvant treatment strategy, since vitamin D may protect against tumor progression through its pleiotropic anticancer effects via binding to its Vitamin D Receptor (VDR) [11].

### Vitamin D status: current knowledge

**Fig. 1.**
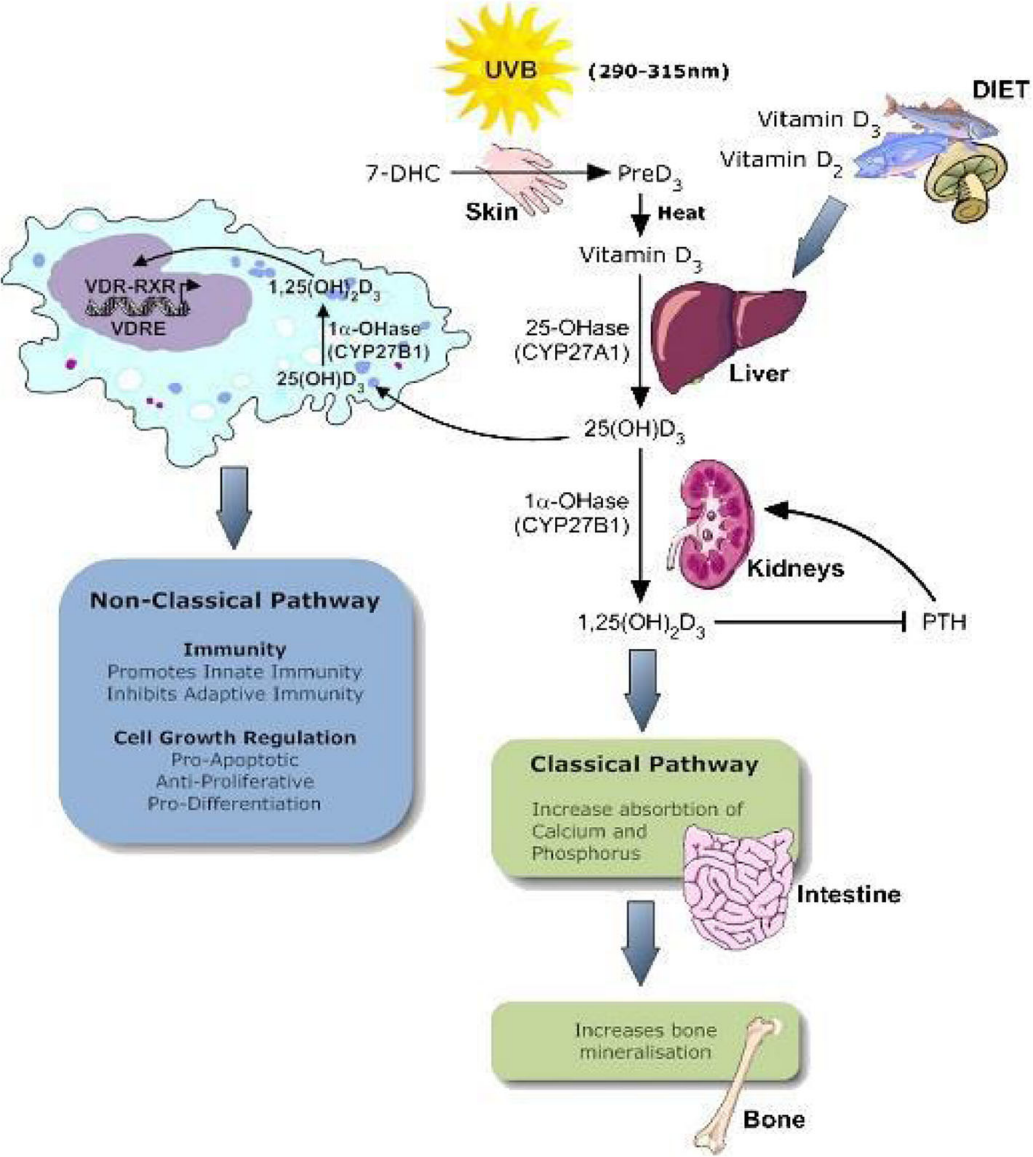
Physiology of Vitamin D, reprinted from Melanoma and vitamin D, Molecular Oncology 5, Field S., Newton-Bishop JA., 2011; 197-214 [11]. Copyright 2016, with permission from Elsevier

Vitamin D, a fat-soluble seco-steroid, has two sources. The main source, vitamin D3 (cholecalciferol), is the skin. The other source, vitamin D2 (ergocalciferol) and D3, is exogenous and is ingested via dietary intake or supplements. The endogenous pathway is the most important source of vitamin D for humans. 7-dehydrocholesterol (7-DHC), a provitamin in the epidermis, is photoactivated by ultraviolet B (UVB) radiation into provitamin D3. A series of modifications in the skin, liver and kidneys metabolizes vitamin D2 and vitamin D3 (dietary and endogenous) into the active substance 1α,25 dihydroxyvitamin D3 (calcitriol, 1,25(OH)2D3). 1,25(OH)2D3 and retinoid X receptor form a heterodimer and have an effect on gene expression by binding to the vitamin D receptor [11]. This is illustrated in Fig. 1.

The active metabolite of vitamin D, 1,25(OH)2D3, demonstrates relevant anti-cancer effects on melanoma cells. It has antiproliferative, differentiating and proapoptotic effects, but also inhibitory effects on tumour invasion and metastasis [12, 13]. These pleiotropic anticancer effects are mediated by the VDR. Serum concentration of 25 hydroxy vitamin D3 (25(OH)D3) is the best indicator of the vitamin D status, which is determined by UVB induced production of vitamin D in the skin, dietary intake and vitamin D supplementation [14]. In addition vitamin D status may also be influenced by genetic variants of certain proteins involved in the vitamin D pathway [15]. A serum concentration of 25(OH)D3 below 10 ng/ml (<25 nmol/L) is considered as vitamin D deficient, and a serum concentration below 20 ng/ml (<50 nmol/L) as vitamin D insufficient. The optimal level for health is believed to be reached at 30 ng/ml (75 nmol/L). Toxic levels are in the range of 100–150 ng/ml (or 250–375 nmol/L). Vitamin D supplementation in the form of vitamin D3 can raise 25(OH)D3 concentrations and is safe when given in a single monthly dose of 100,000 IU during a year and given in an oral loading dose of 500,000 IU followed by an oral dose of 50,000 IU monthly for 2 years [16, 17]. Current recommendation for additional vitamin D intake via supplements is 1000 IU/d. Norman and Bouillon showed a good safety profile for this amount in more than 50,000 subjects over several years [18]. 25(OH)D3 concentrations reach a plateau after 3 months, when vitamin D3 supplementation is given monthly in a dose of 1000 IU/d [19]. There is an established proof of concept that vitamin D has an anticancer effect on CMM.

Already 30 years ago, in vitro studies demonstrated an anti-melanoma activity of 1,25(OH)2D3 with effects on cellular growth, differentiation, apoptosis, malignant cell invasion and metastasis [20]. These processes are possibly being mediated through the expression of VDR in malignant melanoma cells and primary melanoma tissue [21]. In vivo vitamin D has also shown to suppress growth of human malignant melanoma derived xenografts in immunosuppressed mice [22].

Previous studies have investigated the effect of vitamin D serum levels on CMM outcome, at diagnosis and/or during the follow up period after treatment [9, 23]. However conflicting results were obtained from these studies. The study of Newton Bishop et al. indicated that high vitamin D levels at diagnosis may have a protective effect on malignant melanoma outcome [9]. The study of Saiag et al. showed that the variability of 25(OH)D3 during follow-up of melanoma patients, but not the level at diagnosis per se was an independent prognostic marker [23]. A recent study showed that the associations of lower levels of vitamin D with poorer overall survival, melanoma-specific survival and disease-free survival were independent from the association between lower vitamin D and higher C-reactive protein [24].To further elucidate a possible protective effect of vitamin D on melanoma outcome we initiated a multicenter randomized double blind placebo-controlled phase III trial to assess the effect of vitamin D supplementation on CMM relapse in the follow-up period after diagnosis and surgery of the primary tumor.

### Primary objectives

The primary endpoint for this study is relapse-free survival. We will test the hypothesis that vitamin D supplementation after removal of the primary tumor has a protective effect on CMM relapse. We will also assess the immunohistochemical expression of VDR in the primary tumor and its possible correlation with relapse.

### Secondary objectives

Vitamin D levels at diagnosis will be correlated with melanoma site, subtype and stage at diagnosis. We will monitor increases in vitamin D levels after supplementation. This will allow us to assess whether every patient is characterized by the same increase in vitamin D or whether serum levels following intake depend on the genetic variability in the vitamin D pathway and whether this genetic variability correlates with stage at diagnosis.

Furthermore, we will investigate whether VDR immunoreactivity correlates with stage at diagnosis.

## Methods and design

### Study design

This is a double blind placebo-controlled phase III multicenter study conducted at the University Hospitals Leuven, the University Hospitals of Antwerp, the Centre Hospitalier Universitaire de Liège in Belgium and at the Medical and Health Science Center at the University of Debrecen in Hungary.

### Overview of study flow

**Fig. 2.**
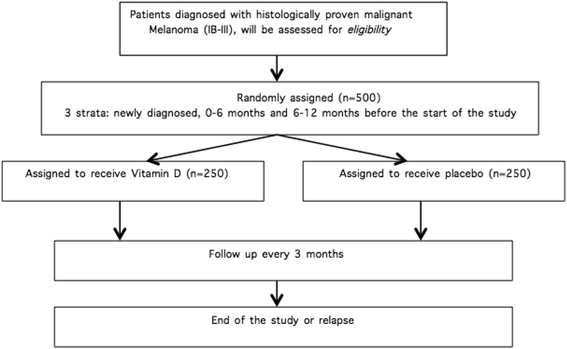
Study flow chart

There is a random assignment of patients in a 1:1 ratio. The treatment group receives a monthly oral dose of 100,000 IU of vitamin D (4 ml of D-Cure™) while the control group receives a monthly oral dose of placebo (4 ml of arachidis oleum). To overcome seasonal influences on baseline characteristics, a block randomization method stratifies on center and the time of diagnosis. Per center there are 3 strata: patients diagnosed within 6 months, between 6 and 12 months before start of the study and newly diagnosed patients (Fig. 2).

### Participants

The aim is to enroll approximately 500 patients between the age of 18 and 80 years old. Eligible participants are patients with histologically proven malignant melanoma at high risk of recurrence namely in stage IB-III (stage IA patients will not be included in the study because they only have a very small chance of relapsing). Inclusion in the study is only allowed after a complete resection of the melanoma has occurred. Participants are included up to 1 year after diagnosis and after signing an informed consent, which had been approved by the local ethics committee. For detailed inclusion and exclusion criteria see Appendix 1.

### Materials

Participants randomized to the active treatment arm, will receive 1 oral syringe filled with 4 ampoules of D-cure™ (Laboratories SMB NV, Brussels, Belgium), corresponding to 100,000 IU cholecalciferol, on a monthly basis. Content of 1 such ampoule is cholecalciferol 25,000 IU, DL-α-tocopherol acetate, sorbitol oleate, orange peel oil and arachis oil ad 1 ml. Participants randomized to the placebo arm, will receive 1 oral syringe containing 4 ml arachis oil (manufactured by Fagron, Waregem, Belgium).

### Study drug administration and randomization

An independent site pharmacist prepares the medication. A treatment and corresponding medication is assigned to the patient during randomization. Medication is taken monthly consisting of either a dose of 100,000 IU (4 ml of D-cure™ suspension in arachis oil) or 4 ml of placebo (arachis oil). The first intake of the drug is given immediately at randomization in the hospital. Subsequent doses are taken monthly at home and are registered in a patient diary. Oral syringes are numbered according to the randomization schedule. At each visit, 3 (if a control is foreseen every 3 months in the university setting) or 6 syringes (if a control is only foreseen every 6 months in the university setting and meanwhile by a peripheral dermatologist) are given to the patient. The date when the vitamin D/placebo is taken and the number of syringes used are registered. Empty syringes are destroyed.

### Screening and randomization of patients

From Q4 2012 to Q4 2016 eligible stage IB-III melanoma patients will be enrolled in our study. After staging, surgical treatment and signing an informed consent form, patients are screened and all in- and exclusion criteria (see Appendix 1) verified. When deemed to be eligible, the patient is randomized by the Leuven Coordinating Centre (LCC, Leuven, Belgium) using an Interactive Voice Response System (IVRS) on an equal basis (1:1) to either vitamin D or placebo group, stratified on time of diagnosis (newly diagnosed, 0–6 months or 6–12 months before start of the study) and centre. All study staff members, trial nurses, dermatologists and patients are blinded to the treatment allocation during the whole study duration. The data management center keeps the randomization schedule confidential. The study complies with the Helsinki declaration of medical research and is approved by the hospital medical ethics committees of the 4 participating centres.

### Study conduct

At randomization a questionnaire is completed for every participant with questions on melanoma risk factors, including the amount and type of sun exposure and a full skin examination for the assessment of phototype, naevus phenotype and actinic damage is completed. Body Mass Index (BMI) is estimated after measuring the patient’s height and weight.

To monitor the effects of high dose vitamin D supplementation, diagnostic tests and assays are performed at regular time intervals. Serum 25(OH)D3 levels are measured at randomization and every 6 months subsequently. Every 3 months and at the end of the study serum calcium and serum phosphate are measured. Renal and liver function tests are evaluated at randomization and at the end of the study. At the end of the study, a 24-h urinary calcium excretion test is done. For detailed information about schedule assessments see Table 1. At randomization, the study team will give the patient a diary in which to record their use of drugs and side effects which are reviewed by the clinician at follow-ups. Dose adherence of the patients is controlled by recording the number of empty syringes which the patients need to bring back. Adherence to the study drug is improved by seeing the patients at regular intervals (3 monthly) either by the general practitioner, private dermatologist or at a university hospital setting. Patients are followed until relapse or end of study.

**Table 1:** Schedule of assessments

	Screening	Randomization	3 monthly FU	6 monthly FU	End of study
25(OH)D3 serum levels		X	X (when 25(OH)D3 levels >80 ng/ml and study drug temporarily interrupted)	X	
Blood sample for DNA analysis		X			
Hemoglobin, Hematocrit	X				X
WBC count and differentiation	X			X	X
RBC count	X				X
Platelet count	X				X
Renal function (creatinine)	X				X
Liver function (AF, ALT, gamma-GT, LDH)	X				X
Serum calcium	X		X		X
Serum phosphate	X		X		X
Albumin	X				X
24 h urine calcium					X

### Safety

Baseline laboratory tests are performed at screening of the patient and safety laboratory every 3 months of follow-up. The parameters that are analyzed can be found in Table 1. Laboratory parameters are only reported as abnormal if the investigator assesses them as ‘clinically significant’ and/or when they result in precautionary safety measures. The following laboratory values will be reported as adverse events (AEs):

- Hypercalcemia: defined as 2 independent measurements above the reference value as defined by the laboratory where the sample is determined (first measurement at the investigational site and second measurement at general practitioner/dermatologist)
- Hypervitaminosis D: defined as serum 25(OH)2D3 level > 80 ng/ml
- Hypovitaminosis D:
  Deficient: serum 25(OH)2D3 < 10 ng/ml
  Insufficient: serum 25(OH)2D3 ≥ 10 ng/ml and <20 ng/ml
- Hypophosphatemia: defined as phosphate level < 1.5 mg/dl
- Hyperphosphatemia: defined as phosphate level > 6 mg/dl

Immunosuppressive effects are evaluated by reporting the rate of infection every 3 months and by measuring the total White Blood Cell (WBC) count and differential every 6 months. For detailed information see Appendix 2. All AEs are documented by study investigators. An AE is defined as any untoward medical occurrence in a patient administered with an investigational medicinal product and which does not necessarily have a causal relationship with this treatment. Severity of AEs is assessed (mild, moderate, severe or unknown). A Serious Adverse Event (SAE) is defined as any untoward medical occurrence that at any dose results in death, is life-threatening, requires inpatient hospitalization or prolongation of existing hospitalization, results in persistent or significant disability/incapacity, congenital anomal/birth defect or is medically significant. SAEs are documented within 24 h in the SAE module provided in the electronic Case Report Form (eCRF) and reported to the LCC. All SAEs are monitored and followed up until the outcome is known. The Data and Safety Monitoring Board (DSMB) reviews all safety parameters and AEs. This board is composed of a dermatologist, an oncologist and biostatistician, who are all not involved in the study and are unblinded. At regular intervals, an unblinded interim analysis is performed to assess the difference between the intervention arms and to exclude an increase in relapse rate in the vitamin D supplemented arm.

### VDR expression in primary melanoma

The expression of the VDR is immunohistochemically assessed in the primary tumor of each melanoma patient that is enrolled in the study. This is done on formalin-fixed paraffin embedded (FFPE) tissue that is collected at diagnosis and is stored according to the standard procedures of the department of pathology (Professor J. van den Oord). FFPE slides from every melanoma are stained on an autostainer (operational) using the Envision technique and a commercial monoclonal anti-VDR antibody. The levels of VDR expression and their localization in the different phases are semi-quantitatively assessed by an experienced pathologist (Dr. *C. aura*).

### Determination of 25-(OH)D serum levels

Serum 25(OH)D3 levels are determined at randomization and every 6 months, using a commercially available radioimmunoassay (RIA) kit: Diasorin, Stillwater, MN (USA) as described [16]. This RIA is well accepted as a reference protocol and commonly used to determine 25(OH)D3 levels in the field. The laboratory of “Experimental medicine and Endocrinology” (Professor D. Vanderschueren) has extensive experience in measuring 25(OH)D3 levels and will therefore perform all these measurements [16]. The intra- and inter-assay coefficients of variation for 25(OH)D are 11% and 8% respectively. The detection of the RIA kit is 5.0 nmol/L 25(OH)D. In addition, serum 25(OH)D3 levels will also be determined with an in house liquid chromatography/tandem mass spectrophotometry method which is currently been developed and validated in the diagnostic laboratory of the Leuven University hospital.

### DNA analysis

A bloodsample for DNA analysis is taken at randomization. Genetic variants affecting vitamin D levels are genotyped by using iPLEX technology. Multiple single nucleotide polymorphisms (SNPs) have been reported to affect vitamin D signaling. We analyse 4 common SNPs (rs12785878, rs10741657, rs6013897 and rs7041), because they have the most important effect on 25(OH)D3 levels. Fifteen candidate SNPs, located in genes in the vitamin D pathway and previously reported to affect vitamin D signaling, are selected as well. Those 15 SNPs may independently determine 25(OH)D3 levels.

### Participant follow-up

On a 3 monthly basis, there is a follow-up of all patients included in the study. Consisting of physical examination, including full skin assessment and palpation of regional lymph nodes and blood sample tests (see Table 1). At intervals of 6 months (or upon clinical indication), an ultrasound of the lymph nodes will be performed. Only when new symptoms arise or physical findings are suggestive for progression, a Positron Emission Tomography-Computed Tomography (PET-CT) or Magnetic Resonance Imaging (MRI) is performed to exclude distant metastasis. Every 12 months, an ultrasound of the abdomen and x-ray of the chest (or CT thorax/abdomen if clinically indicated) are performed.

### Blinding

Both patients and investigators are blinded to study medication and vitamin D levels.

### Study duration

Patients enrolled in the study will be followed up throughout the entire study duration, with a maximum of 3,5 years follow-up. Participation of a patient may be terminated earlier due to relapse/progression or due to a reasonable cause, such as the investigator’s medical decision. At any time, the patient has the right to withdraw consent without a negative impact on her/his medical treatment. The planned enrolment rate is 500 melanoma patients during a recruitment phase of 4 years maximum. The first patient was recruited in fall 2012 and the last patient will be recruited in fall 2016. The study will stop 6 months after the last patient is randomized. The study may terminate earlier due to interruption of the DSMB, the sponsor, competent authorities/ethics committees or if the work is not compliant with Good Clinical Practice.

### Sample size justification

Assuming similar times to relapse as retrospectively observed at the University hospital Leuven, 3 years of recruitment and a total study duration of 3.5 years, 500 patients will have 90% power to detect a hazard ratio of 0.40 in favour of the vitamin D supplemented arm by means of Cox proportional hazards model stratified for time since diagnosis (3 strata: 0, 0–6 or 6–12 months ago). The hazard ratio of 0.40 is in correspondence with the intermediate effect size that was reported by Newton-Bishop et al. [9] and a potential increase in VD serum levels of 70 nmol/L, which is 80% of the observed effect in a study with COPD patients [16]. It was anticipated that no patient will be lost for follow-up. Due to a slow recruitment, the recruitment period was extended by 1 year.

### Statistical methods

The primary analysis is an intent-to-treat analysis of all randomized patients. Patients will be analysed according to which treatment group they were randomized, irrespective of which study drug or even if any study drug was received. A Cox proportional hazard model with stratification for time since diagnosis (3 strata: 0, 0–6 or 6–12 months ago) will be performed to investigate the difference between the two treatment groups for the primary endpoint, which is relapse-free survival. If the treatment effect is stratum dependent, then the strata will be analysed separately. A correction for baseline covariates like sex, age and the baseline vitamin D level will be performed as sensitivity analyses. A Cox proportional hazard model will also be used to investigate whether a potential difference in relapse-free survival between two treatment groups is different for VDR immunoreactivity and genetic variability in the vitamin D pathway. The hazard ratios and associated 95%-confidence intervals (CI) will be determined to assess the contributions of significant factors. All reported *p*-values will be two-sided, and a *p*-value less than 0.05 will be considered statistically significant. A linear mixed model will be used to identify the evolution of 25(OH)D3 levels in function of genetic variability in the vitamin D pathway. In order to examine the correlation between VDR immunoreactivity and stage at diagnosis, a Kruskal-Wallis test will be used. For newly diagnosed melanoma patients, an analysis of variance will be performed to correlate vitamin D levels at diagnosis, melanoma site and subtype. Full details of the statistical analyses will be described in a statistical analysis plan which will be finalized before database lock.

### Quality assurance

All efforts are being made to reassure maximal quality control. The study is performed according to good manufacturing practices. Direct access to source data, with strict adherence to all confidentiality regulations, is available for monitoring purposes. In addition, a DSMB reviews all safety parameters and AEs.

## Discussion

To the best of our knowledge, this is one of the first randomized, placebo-controlled phase III trial to examine the efficacy and safety of long-term and high-dose vitamin D supplementation in melanoma patients, with a maximum time of supplementation of 3,5 years [17]. Patients are randomized in a 1:1 ratio, either receiving 100,000 IU cholecalciferol or placebo. The patients’ clinical status, including adverse events, is monitored at regular intervals. Blood tests and other technical investigations are also regularly conducted as explained previously (see Table 1). There is a rising interest in vitamin D with respect to its pleiotropic effects on chronic diseases and cancer. The objective of the project is to build further on the knowledge of these effects in the field of melanoma treatment. Keeping in mind, the compromised prognosis of patients with relapsing malignant melanoma and the health as well as economic burden associated with metastatic disease we consider vitamin D supplementation a considerably save, low cost and broadly acceptable adjuvant strategy worthwhile being tested. If we can confirm our hypothesis that vitamin D supplementation after removal of the tumor has a protective effect on relapse of CMM, we may markedly reduce the burden of CMM at several levels. Patients, diagnosed with melanoma may have a better clinical outcome, which not only means a decrease in loss of years but also a decrease in loss of professional years (decreased mortality in still professionally active young population). Patients diagnosed with melanoma will have an improved quality of life (less relapse, less complications due to treatment of regional metastatic and distant metastatic disease). Finally, there will be a decrease in health care costs related to treatment of regional and distant metastatic disease.

## Registration

The study is registered with Clinical Trial.gov (NCT01748448). Eudract No: 2012–002125-30.

## Protocol

A full copy of the protocol of the current ViDMe study can be requested from the principal investigator Prof. Dr. Marjan Garmyn, email: marjan.garmyn@kuleuven.be.

## Appendix 1

Inclusion and exclusion criteria for the ViDMe study

### Inclusion criteria

1. Older than 18 years and younger than 80 years of age.
2. Histologically proven malignant melanoma, stage IB to III. Not participating in another clinical trial.
3. The only treatment for melanoma is surgical treatment.
4. Complete resection of melanoma.
5. Single primary invasive cutaneous melanoma (inclusion within 1 year after diagnosis).
6. Signed ethical committee approved informed consent.
7. Serum phosphate and calcium at the entry of the study within normal range of the laboratory reference.

### Exclusion criteria

1. Pregnant/lactating women or planning on becoming pregnant during the study.
2. Known hypersensitivity to vitamin D or its components.
3. Pre-existing renal stone disease or chronic renal disease.
4. Impaired renal function with an eGRF <30 mL/min/1.73 m2 or renal dialysis.
5. Liver failure or chronic liver disease with liver enzyme >2 fold the upper limit of normal.
6. History of parathyroid disease or granulomatous disease (tuberculosis or sarcoidosis).
7. History of malabsorption syndrome or any medical condition that might interfere with Vitamin D absorption.
8. History of other malignancy within the last 5 years except for carcinoma in situ of the cervix or basal cell carcinoma or squamous cell carcinoma of the skin or in situ malignant melanoma.
9. Chronic alcohol abuse.
10. Medical or logistic problems likely to preclude completion of the study

## Appendix 2

Safety procedures in the event of abnormal assay results

### End of study

- Clinical significant hypercalcemia: defined as 2 independent measurements above the reference value
- Hypovitaminosis D (serum 25(OH)2D3 < 10 ng/ml)
- Pregnancy
- Disease progression

### Action taken

- Hypervitaminosis D: Defined as 25(OH)2D3 > 80 ng/ml. Discontinue study drug and restart monthly treatment with study drug when 25(OH)2D3 falls below 50 ng/ml based on a 3 monthly monitoring.
- Hypovitaminosis D: serum 25(OH)2D3 > 10 ng/ml and <20 ng/ml: supplementation of extra vitamin D (D-cure): 25,000 IU/month
- Hypophosphatemia: < 1.5 mg/dl: further investigation
- Hyperphosphatemia: > 6 mg/dl: further investigation

### Concomittant medications/treatments

Concomitant use of vitamin D or multi-vitamin supplements, concomitant use of calcium supplements and medications which we know interact with study medication are registered.

Other medications then listed below, will not be recorded:

- Digoxin
- Lithium
- Thiazide diuretics
- Anticonvulsants
- Corticosteroids (Chronic oral use is an exclusion criteria, topical use is permitted)
- H2 receptor antagonists

## Abbreviations

1,25(OH)2D3: 1α,25 dihydroxyvitamin D3
25(OH)D3: 25 hydroxyvitamin D3
7-DHC: 7-dehydrocholesterol
AE: Adverse Event
AF: Alkaline phosphatase
AJCC: American Joint Committee on Cancer
ALT: Alanine Aminotransferase
BMI: Body Mass Index
CMM: Cutaneous Malignant Melanoma
COPD: Chronic Obstructive Pulmonary Disease
CTA: Clinical Trial Assistant
DSMB: Data and Safety Monitoring Board
eCRF: electronic Case Report Form
FFPE: Formalin-Fixed Paraffin Embedded
Gamma-GT: Gamma-Glutamyl Transferase
IU: International Unit
IVRS: Interactive Voice Response System
LCC: Leuven Coordinating Centre
LDH: Lactaat Dehydrogenase
MRI: Magnetic Resonance Imaging
PET-CT: Positron Emission Tomography–Computed Tomography
RBC: Red Blood Cell
RIA: Radioimmunoassay
SAE: Serious Adverse Event
UVB: Ultraviolet B
VDR: Vitamin D Receptor
WBC: White Blood Cell Count
